# Opportunities for Smartphone Sensing in E-Health Research: A Narrative Review

**DOI:** 10.3390/s22103893

**Published:** 2022-05-20

**Authors:** Pranav Kulkarni, Reuben Kirkham, Roisin McNaney

**Affiliations:** Department of Human Centered Computing, Faculty of IT, Monash University, Clayton, VIC 3168, Australia; reuben.kirkham@monash.edu (R.K.); roisin.mcnaney@monash.edu (R.M.)

**Keywords:** smartphone sensing, digital health, e-health, narrative review

## Abstract

Recent years have seen significant advances in the sensing capabilities of smartphones, enabling them to collect rich contextual information such as location, device usage, and human activity at a given point in time. Combined with widespread user adoption and the ability to gather user data remotely, smartphone-based sensing has become an appealing choice for health research. Numerous studies over the years have demonstrated the promise of using smartphone-based sensing to monitor a range of health conditions, particularly mental health conditions. However, as research is progressing to develop the predictive capabilities of smartphones, it becomes even more crucial to fully understand the capabilities and limitations of using this technology, given its potential impact on human health. To this end, this paper presents a narrative review of smartphone-sensing literature from the past 5 years, to highlight the opportunities and challenges of this approach in healthcare. It provides an overview of the type of health conditions studied, the types of data collected, tools used, and the challenges encountered in using smartphones for healthcare studies, which aims to serve as a guide for researchers wishing to embark on similar research in the future. Our findings highlight the predominance of mental health studies, discuss the opportunities of using standardized sensing approaches and machine-learning advancements, and present the trends of smartphone sensing in healthcare over the years.

## 1. Introduction

Over the past decade, there has been a substantial number of technological advancements, especially in terms of the availability of mobile sensing on smartphones. Mobile-phone technology in particular has witnessed tremendous growth, both in functionality and consumer adoption [1]. Traditional mobile phones of the early 2000s have been superseded by smartphones across the world; these offer a greater variety of features beyond calling and messaging, such as browsing the internet and capturing audio/video content. Importantly for eHealth studies, they also include a suite of sensors, which have the capability to capture human behavior and the ability to transmit data collected.

This has been a result of both hardware and software advances, which have led to improvements in performance and power consumption. Devices today are embedded with a rich set of low-power sensors, which can passively record contextual user data. For example, accelerometers are commonly used in smartphones to record the acceleration forces acting upon the device, whereas light sensors are used to measure the amount of light the device is exposed to. While these sensors facilitate data collection, software frameworks facilitate the transformation of this raw data into meaningful insights relevant to human health and wellbeing. If we take the accelerometer as an example, data extracted from this sensor can be used for activity recognition, such as providing an estimate of the steps taken by the user, which might indicate general activity levels of the user [2,3]. Data from the light sensor (which is generally used for the screen auto-brightness feature of smartphones) could give an indication of the time spent in a dark room, which could potentially indicate a decline in the mood or mental health of a user [4,5]. Most smartphones also have the potential to track additional usage statistics, such as screen time, application usage, and battery usage; as such, they have the capacity to capture a large amount of user information and are becoming increasingly context-aware.

In addition, users spend a considerable amount of time using their phones. According to a survey study conducted in February 2021 [6], 46% of US adults stated that they use their phones for an average of 5–6 h per day. The number of hours users spend using their phones, combined with the rich data and user context that is captured by the phones, can provide significant insights about a person. For example, the Global Positioning System (GPS) data collected can provide a snapshot of the places a person visits in a day [7,8,9]. Combined with human activity recognition (“HAR”) algorithms, one could identify how the person commutes to these places—walking, running or by using a vehicle—as well as indicating their level of physical activity [3,10,11]. By combining the insights gained from all of the sensors, one can glean a range of insights about a person’s daily activities. Thus, smartphones to some extent can act as a proxy data source, providing information about a person that would otherwise require significant human effort to collect (e.g., through paper or electronic diaries).

These capabilities, high user adoption, and the relatively unobtrusive nature of smartphones make them an appealing choice for ‘in-the-wild’ healthcare studies [3,10,12,13]. Several studies have utilized smartphones to remotely collect data from users and gain health insights from these data. Studies have explored how smartphone-based sensing can be used to infer mood [14,15,16,17,18,19,20,21], physical activity [3,11,22,23], and mental health [13,24,25,26,27], and also to track the effects of chronic health conditions [28,29,30,31]. Moreover, software frameworks have also been developed to help researchers use smartphone sensing to conduct their own studies, such as AWARE [32], Beiwe [33], and EARS [34] to name a few. These offer considerable promise for the future of eHealth research and, in turn, improved opportunities and outcomes for patients.

At present, there is a significant amount of multidisciplinary work being carried out in the space of smartphone sensing, with diverse motivations, methods, and approaches being used by health practitioners, HCI researchers and computer scientists. The sheer number of studies and different approaches can make it challenging for researchers to evaluate the appropriateness of smartphone-based sensing for different use cases. In this paper, we analyze the existing literature in this space to address this issue and provide a high-level overview of the various use cases, opportunities, and challenges of smartphone sensing in healthcare. After setting out our literature-selection approach (Section 2), we present a full account (Section 3) of the various health conditions studied, types of data collected, tools used, and barriers identified in the literature, which will benefit future researchers by helping them to make high-level decisions regarding different approaches for future studies using smartphone sensing. We then present the wider discussion points (Section 4), including our specific findings: (1) predominance of mental health studies, (2) opportunities for standardization of sensing approaches, (3) opportunities for using machine-learning advancements in eHealth research, and (4) sensing trends over the years and future scope. The discussion is followed up by limitations and future work (Section 5), with the last section being the conclusion (Section 6).

## 2. Materials and Methods

The main goal of this study was to understand the potential of smartphone sensing in healthcare, with a view towards providing a full understanding of existing practice. As such, the review aimed to understand the following high-level concerns:

- What health conditions have been examined using smartphone sensing?
- What data-collection approaches have been used and what are their trade-offs?
- What applications and resources have been used?
- What were the researchers’ motivations for their data-collection and -analysis approaches?

To address these aims, our work utilizes a narrative literature-review methodology. Narrative literature reviews (NLRs) provide a broad overview of a given topic by synthesizing previously published literature. They are valuable, as they condense information from a variety of sources and provide an understanding of the domain without having the reader interrogate the entire literature [35]. There are a few key reasons for choosing this methodology over a systematic literature review, which is the approach typically followed in the clinical literature. Firstly, the field of smartphone sensing is very broad and there are numerous subdomains in this space, such as human-activity recognition, behavior prediction, and self-tracking to name a few. This work aims to present a broad perspective of all such areas to provide insights for new researchers in eHealth around the opportunities and challenges in this specific context.

Secondly, there are numerous systematic reviews that provide a comprehensive insight on smartphone sensing for various specific health conditions [31,36,37,38], smartphone-based activity recognition [39,40], and sensing apps and datasets [41]. Rather than replicating these and providing a quantitative insight into the fields, a narrative review allows for a qualitative approach to highlight overall advancements. This review aims to create new knowledge by synthesizing the insights from all these subdomains, thus providing a synopsis of current practices, to aid in the design and deployment of future smartphone-based health studies.

Whilst systematic reviews have guidelines such as the PRISMA (Preferred Reporting Items for Systematic Reviews and Meta-Analyses) [42] that discuss the methods and reporting the findings, no such formal specifications exist for NRLs. However, a few authors have provided a set of recommendations to follow while writing [35,43] and evaluating [44] NRLs. This review follows these recommendations by systematically discussing the literature search terms, search strategy, and inclusion/exclusion criteria.

### 2.1. Search Terms

The multidisciplinary research team (with expertise in chronic and mental health conditions, disability, assistive technologies, smartphone and physical sensing, and ubiquitous computing) met on multiple occasions to iteratively brainstorm and decide on a set of search terms to explicitly explore how smartphone sensing has been used in the health space. Four keywords were identified, which were (1) “smartphone sensing”; (2) “self-tracking” AND “smartphone sensing”; (3) “quantified self” AND “smartphone sensing”; (4) “mobile sensing” AND “human health”. As we were interested in exploring smartphone-sensing research across the domain of human health (and not focused on a specific condition), these search terms were selected to encompass the breadth of smartphone-sensing literature, allowing for further assessment of the relevance of articles in the human health space, while restricting articles discussing other aspects of mobile sensing and wearables. The fourth search term was selected because mobile sensing is analogous to smartphone sensing, but the additional keyword was added to make the search more relevant to the area of focus.

### 2.2. Search Strategy

Two databases were selected for the search, Google Scholar and PubMed. Given the rapid development in smartphone technology and software regulatory changes impacting sensing approaches [45,46], the search was restricted to the past 5 years (2017–2022), to discover the latest developments in the space. The four keywords were then individually used to find literature on the two databases. The titles of the search results were read and a list of potentially relevant peer-reviewed, full-text articles in English was created. After the removal of duplicate articles, the abstracts were read to evaluate whether the article met the inclusion criteria.

### 2.3. Inclusion Criteria

The goal of this literature review was to understand the opportunities and challenges of smartphone sensing in health studies. As a large population over the globe has access to smartphone technology [1], the aim was to explore the feasibility of smartphones as a standalone device in a ‘real-life’ scenario. As such, only studies that discussed smartphone sensing were considered, and literature using body-worn sensors was excluded. Articles were selected if they met the following inclusion criteria:

1. Empirical research that uses smartphone sensing to monitor a health condition;
2. Empirical research that explores perceptions and challenges of smartphone sensing.

The initial literature search provided a total of 3080 hits: 3034 from Google Scholar and 46 from PubMed. After reading the titles for relevance (and excluding duplicates), a total of 222 articles were present. The abstracts of each of these were read to determine if they met the inclusion criteria. For example, although [47] used a smartphone-sensing app to monitor user behavior, the aim was to understand compliance to COVID-19 policies and was thus excluded. Another exclusion example was a study examining smartphone-based self-tracking adherence for chronic health conditions [48]. Although the study used a smartphone self-tracking app, it also allowed the participants to import data from a wearable device and was thus excluded. After the removal of such examples, a total of 86 articles were present.

## 3. Results

Out of the 86 articles, 71 were empirical studies using smartphone sensing to monitor health conditions; 9 were explicitly discussing the technical and ethical challenges of smartphone sensing; and 6 papers were discussing user perceptions of smartphone sensing. The challenges, user concerns, and opportunities that were discussed in the 15 papers have been described in various sections. The results section first discusses the various health conditions that have been examined, followed by the data-collection approaches used. We then discuss the frameworks used to facilitate data collection, and lastly, the purpose of the data collection (i.e., what the authors were trying to find out in their studies).

### 3.1. Health Conditions That Have Been Studied

**Figure 1:**
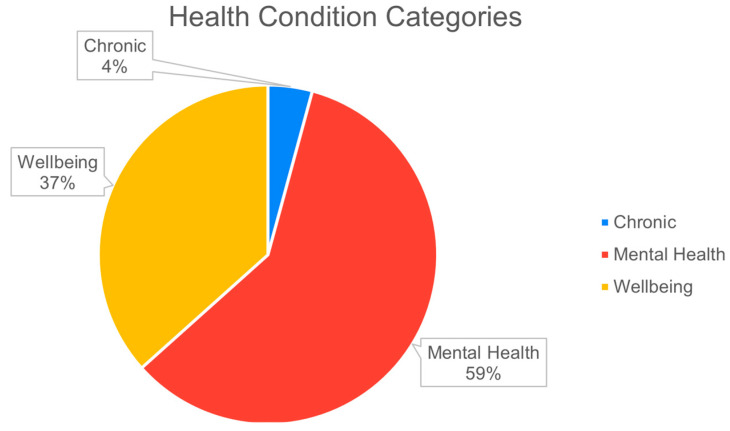
Types of conditions studied using smartphone sensing.

Smartphone sensing has been used to study a wide range of conditions. Not only have studies used smartphone sensing to evidence factors impacting physical wellbeing, but they have also been used to obtain insights about mental health. The health conditions can be categorized into three categories: general wellbeing, chronic health conditions, and mental health conditions. From the 71 empirical studies, mental health conditions were by far the most common (42 articles), followed by general wellbeing (26 articles) and lastly chronic conditions (3 articles), as shown in Figure 1.

**Figure 2:**
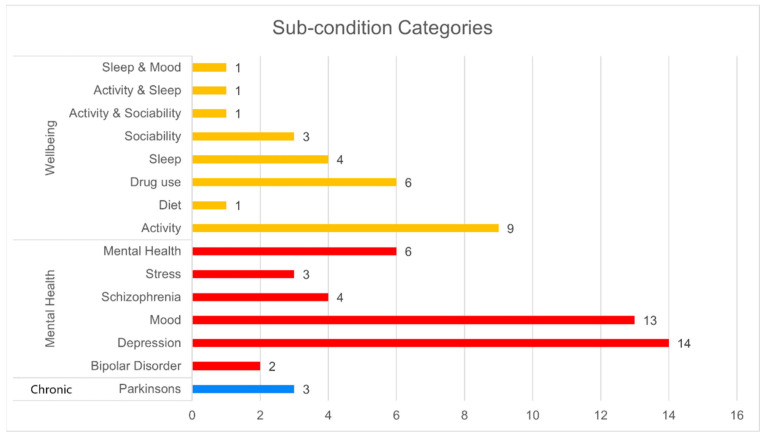
Specific subconditions studied using smartphone sensing.

Under each of these categories, several specific subconditions were studied. Figure 2 presents an overview of the subconditions, which are discussed in more detail in the following sections.

#### 3.1.1. General Wellbeing

This category discusses various aspects that have an overall impact on wellbeing. The factors that were studied include physical activity, sociability, sleep, diet, and substance use (alcohol, tobacco, cannabis).

The most common factors examined were physical activity/mobility [2,3,10,11,22,23,49,50,51,52,53] and sociability [3,9,54,55]. Sensor data from the accelerometers, gyroscope, and location data (using GPS, Bluetooth) were used to infer factors such as places visited [50], amount of time spent at home [22], types of activity performed (sitting, standing, walking, running) [11] and regularity of movement [3]. On the other hand, location data [53], frequency and duration of social media app use [55], microphone recordings [3] and phone-call and text-message logs [54] were used to provide information about a person’s physical and virtual sociability.

Six studies collected and examined smartphone data as a proxy indicator of a person’s sleep [19,51,56,57,58,59]. Given that a smartphone cannot directly record a person’s sleep, studies used a combination of passively sensed data and user reports to detect sleeping behaviors. Identifying smartphone-usage behaviors during bedtime, such as putting the phone on charge [58] and lack of screen activity [19] and sound [57] assisted in estimating the user’s sleeping behavior.

A further six studies examined the use of smartphone sensing to detect intake and intoxication due to substances such as alcohol [60,61,62], tobacco [63], and cannabis [12,64]. They utilized location and activity data to understand the contexts of consumption and its impact on mobility. For example, cannabis intake would impact gait and reduce physical activity, which can be monitored through accelerometer [64] and location data [12]. This ability to detect intake instances was seen to provide useful opportunities for intervention using smartphones [63].

Finally, one study investigated smartphone-usage behaviors related to food consumption [65]. They explored whether factors such as sociability, location, and activity could be used to infer food-consumption levels of college students and detect episodes of overeating.

#### 3.1.2. Chronic Health Condition (Parkinson’s)

Chronic health conditions have a long-lasting impact on a person’s health and require ongoing medical support [66]. Parkinson’s disease [28,29,30] was the only condition that was discussed in the articles (another study worked with people with diabetes but focused on symptoms of depression [67]). These studies explored the use of smartphone sensing to monitor gait (manner of walking) [28,29,30], posture [28], and voice [30]. Accelerometer and gyroscope data were commonly used in all of the studies, as they examined movement-related symptoms. Additionally, Ref. [30] explored the impact of medication intake on voice by using the microphone sensor.

#### 3.1.3. Mental Health Conditions

The majority of articles (59%) used smartphone sensing to infer mental health conditions. While six articles examined overall mental health [13,24,25,26,27,68], other studies examined specific factors such as mood [15,16,17,18,19,20,21,69,70,71,72,73,74,75] and stress [76,77,78]. Additionally, studies also examined specific mental health conditions such as depression [7,8,67,79,80,81,82,83,84,85,86,87,88,89], schizophrenia [90,91,92,93], and bipolar disorder [94,95].

Given that physical activities and general wellbeing have an impact on mental health, there was some overlap in the type of data used. Studies in this category used a variety of smartphone data, ranging from sensor data (e.g., accelerometers, light sensors) to software features such as application usage, phone and message logs, etc. Such data, along with self-reports, was used to infer the mental health of the users. For example, both [84,86] used microphone-sensor data combined with self-reports to infer depressive behavior by measuring symptoms of depression such as decreased sociability and disturbed sleep. By combining passively sensed data and different forms of user self-reports (e.g., clinical questionnaires [67,79,86] and ecological momentary assessments [16,70,95]), smartphone sensing was widely used to monitor and predict the mental wellbeing of individuals.

### 3.2. Data-Collection Approaches

This section discusses the different approaches and types of data collected by the studies. Data collection was conducted using two approaches: actively collected data and passively sensed data. As the name suggests, active data collection involved collecting user input and relied on user compliance. On the other hand, passive data collection used the embedded device sensors to collect user data and relied less heavily on user input and compliance. The majority of the 71 studies (84.5%) used both types of data collection, while the remainder used either active (7%) or passive (8.5%) data. The types of active and passively collected data are discussed in more detail in the following subsections.

#### 3.2.1. Actively Collected Data

Active data collection requires regular user input and relies on user compliance. It requires users to ‘track’ or ‘log’ one or more factors about their health, thus forming a data source, which is then analyzed to obtain insights. The ability to set up schedules and prompt users for input facilitates active data collection. Although these types of data are not sensed and collected directly via user input, they are crucial to consider as they provided useful context to passively sensed data. Studies relied on actively collected data to understand factors that could not be sensed directly (such as mood and mental health) and to label passively collected data as a means to obtain ‘ground truth’ (e.g., users self-labeling their mobility activities to assist in activity-recognition systems). Most papers (91.5%) factored in some level of active data collection and collected different types of data from the users. The types of data collected were as follows:

1. Demographic information

As a one-off means of data collection, studies collected demographic data from the participants prior to study commencement via smartphone (e.g., [3,55,62]). Information such as age, sex, ethnicity, education, etc., were collected, which assisted in understanding the participants and contextualized findings. The vast majority of studies incorporated demographic-information collection.

2. Clinical scale/questionnaire responses

Self-collected clinical questionnaires were commonly administered via smartphone to assess factors related to the user’s wellbeing. They include a set of questions which can evaluate health conditions and their severity. Such questionnaires are validated by the medical community and have proven to be reliable indicators of health factors.

Studies used different clinical questionnaires based on the health condition being studied. For example, most studies monitoring depression used the Patient Health Questionnaire (PHQ) [67,79,80,86], which is used to measure depression severity [96]. Another example is that of the UCLA Loneliness Scale [97], which was used by studies monitoring sociability of the participants [22,27]. There are numerous other scales available, which could potentially be used in future studies.

3. Ecological Momentary Assessments (EMA)/self-reports

The majority of the studies used some forms of Ecological Momentary Assessments to collect self-reported user data. Ecological Momentary Assessments involve repeatedly collecting real-time user information in natural environments [98].

Studies collected information about a variety of metrics to obtain momentary insights about the users. These included factors such as food intake [65], perceived loneliness [9], mood [16,70,95], and stress [22,70], to name a few. Although the questions are not standardized across studies (unlike clinical questionnaires), these too are useful in understanding the user contexts. Moreover, self-reports have been used to add additional context by ‘labeling’ passively sensed data and obtaining ground truth [11,53,57,88], which aids in prediction processes using machine learning.

To summarize, active data collection has been used in most studies and enables the collection of data that may be difficult to interpret from passively sensed data. Table 1 highlights the advantages and disadvantages of active sensing.

#### 3.2.2. Passively Sensed Data

The majority of studies (93%) utilized passively sensed data, by using smartphone sensors for data collection. While there were a few common in-built hardware sensors such as the accelerometer [3,11,53] and GPS/location sensor [9,60,69] used in most studies (62% and 53%, respectively), some also looked at software features such as application usage [61,91]. Studies used a range of sensors and software features to collect various types of contextual information. Table 2 describes the various sensors used, their sensing capabilities, and the type of inferences that have been made from collected data.

Besides these conventional types of sensor and software features, several used less common approaches to infer health conditions. For example, two studies used custom keyboards to collect keystroke/keypress data—one to analyze the sentiment of typed text [78], and the other to infer alcohol intoxication (greater duration between consecutive keypresses, more text deletions and insertions) [62]. One study used the barometer sensor for activity recognition and found that it reduced the misclassification of stair-climbing/descending activity [11]. One study examined the correlation between internet-usage data and PHQ (patient health questionnaire) scores, which provide an indication of depression [87]. Finally, one study explored if social media data (Twitter and Instagram) could provide insight into the mental health of individuals [17].

The potential of smartphone sensing is evident from the wide range of sensor data used in the studies. However, a few important considerations were discussed when deploying passive sensing. These are as follows:

1. Trade-off between power consumption and data-collection rate

Although the wide range of available sensors facilitate diverse data collection, the types of sensors used and the data-collection rate influence power consumption. A higher sampling rate provides larger amounts of data points, but also has a greater impact on battery life [3,53,101]. It is crucial to ensure that the power consumption of the sensing application is minimal, to ensure user acceptance [15,99,101,102]. To minimize the impact on the battery, studies reduced the rate at which data were collected to ensure that their applications were able to collect data for extended periods of time [3,53,84,86]. Moreover, few studies compared the power consumption to other commonly used applications to ensure that it is within acceptable standards [15,67]. As such, this is an important trade-off to consider when using passive sensing.

2. Placement of device

The placement of the device largely influences the accuracy of passive data collection. In contrast to active data collection, where the user provides data when the device is with them, this may not always be the case with passive sensing. There may be circumstances where the users might not carry their devices with them or might not carry them as intended [2,3,11,103]. For example, users may not carry their devices at all or keep their devices in handbags, which may affect the accelerometer and gyroscope readings, leading to inaccuracies in human-activity recognition systems [2,11]. Another example (using the light sensor) might be in situations where the device is kept facing downwards, which would give inaccurate readings of users’ surrounding light conditions [19,84]. Such factors must be accounted for while setting up passive sensing.

3. Data storage and transmission.

Data collected by sensors is naturally required to be stored, processed, and analyzed to obtain insights. Deciding whether the data would be stored locally on the device or transmitted to an external database is another factor to consider [101]. For example, one study that performed real-time analysis stored the data locally on the device [57], whereas other studies temporarily stored data locally and uploaded it to external servers when a Wi-Fi network was available [11,52,53] to reduce data costs. Data storage and transmission costs are important considerations for user acceptance [99].

4. Device operating system

**Figure 3:**
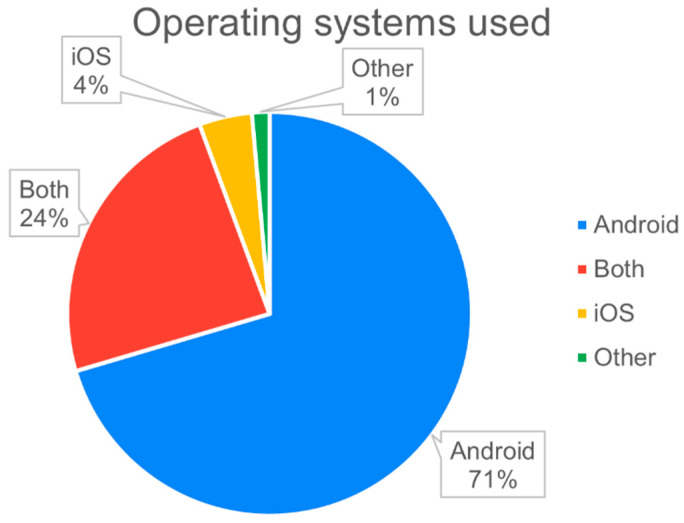
Types of operating systems used in the studies. ‘Both’ refers to the study being conducted on iOS and Android.

The availability of different sensors/software features is dependent on the underlying operating system (Android, iOS). Both systems have different policies in terms of data collection and access. The iOS platform has stricter data-protection regulations and prohibits the direct collection of certain types of data [55]. Studies discussed limitations on collecting data from iOS such as application usage [54,55], GPS and Bluetooth [8,9,88], keyboard presses [62], and phone-call/text-message logs [54,55]. Given such restrictions, it was unsurprising that the majority of the articles used Android devices for their study. Figure 3 presents the distribution of operating systems used in the studies. (Note: one of the studies did not specify the platform that was used [68]).

5. Privacy concerns.

Given the variety and volume of data collected, it is essential to ensure data privacy and confidentiality. Although the topic of privacy in mobile sensing is a whole issue in itself [99,100,104,105,106,107,108], few studies have discussed approaches to minimize privacy invasion of users. Data such as audio recordings from the microphone, keyboard presses, and content of text messages are some examples of particularly sensitive data. For example, studies using the microphone sensor avoided collecting raw, continuous audio data. They collected limited samples to infer higher-level attributes such as amplitude levels [69,84] and presence/absence of voice [3,84,86]. Similarly, the study using keyboard presses only collected every third word and did not collect any passwords/credit card information [78]. Such approaches should be considered to minimize privacy of the users, which is a key factor for user participation and acceptance [99,107].

To summarize, passive sensing has considerable potential for objective data collection while minimizing user burden. However, the aforementioned factors are crucial considerations while collecting passive data.

### 3.3. Applications, Frameworks, and Resources Used in the Studies

Twelve studies discussed the use of existing datasets to test different machine-learning models and algorithms (e.g., StudentLife dataset [27,76,80], mPower dataset [28,29], UCI machine-learning repository [49]). These papers did not collect any primary data during the course of their research.

The majority of researchers, however (41 studies), did collect data and developed their own bespoke applications for the collection and analysis [15,23,55,67,69]. These ranged from apps collecting data from a single category of sensors [23] (e.g., accelerometer, gyroscope for activity recognition) to apps collecting data from both sensor and software features (e.g., app usage [55], battery status [57]). On the other hand, several studies used third-party software and services for data collection, which had similar functionality as the developed applications. Examples of such (paid) services were MovieSens XS [109] and BeHapp [110], used in [93,94], respectively. Ten studies used existing open-source frameworks to develop their applications (see Table 3).

### 3.4. Motivations for Data-Collection and -Analysis Approaches

The studies reported several motivations for data collection, which governed the analysis approaches that were used. There were five common motivations identified:

1. Exploratory studies:
  Four studies were exploratory, presenting the design of their sensing systems and evaluated the data-collection capabilities of their applications. For example, ref. [58] presents a nonobtrusive sleep-detecting application and evaluates how reliably it could detect sleeping behaviors.
2. Monitoring change in behavioral patterns:
  In seven studies, the emphasis was to monitor human behavior using smartphone-sensed data. For example, Refs. [24,25] monitored changes in mental health and behavior during the COVID-19 pandemic, by examining changes in smartphone-sensed data. They examined how factors such as physical activity, sociability, and mobility of students changed due to the pandemic, which provided an indication of their mental health.
3. Identifying correlation between smartphone-sensed features and wellbeing factors:
  In 24 studies, the emphasis was to examine the statistical significance of features extracted from smartphones with wellbeing behaviors. For example, Ref. [86] collected data from the microphone sensor to evaluate if audio features were correlated to self-reported measures of depression. In another example, Ref. [7] collected location data to determine if there was a correlation between time spent at home and self-reported depressive symptoms.
4. Identifying feature correlations and using machine learning to predict behavior:
  These types of studies (22 studies) not only identified correlation between smartphone-sensed features, but also built machine-learning models to evaluate if these were able to predict user behavior. For example, Ref. [60] found location and activity features that correlated with drinking episodes. They then built a machine-learning framework to classify instances of drinking vs nondrinking and tested the performance of their system.
5. Comparing activity-recognition performance of machine-learning models:
  Such studies aimed to evaluate the activity recognition of different machine-learning models. For example, Ref. [49] the performance of five types of ensemble classifiers to classify six activities (walking, walking upstairs, walking downstairs, sitting, standing, and lying).

Given such motivations, many of the systems inherently used some form of machine-learning pipelines, more specifically human-activity recognition (HAR). Across the systems, there were a wide variety of different sensor combinations used, with individual systems aimed at assessing a different range of conditions. However, there was considerable commonality in the underlying approach, with several works (e.g., [11,49,77]) implementing some version of the standard pipeline for HAR systems: (1) data preprocessing, (2) data segmentation, (3) feature extraction, and (4) model training and classification (as explained in [114]), albeit using different classifiers and feature representations. There was, however, a small minority who employed more modern deep-learning approaches, such as neural networks [21,30,53,65]. Overall, only a few studies used more recent approaches of HAR, a matter which we address in more detail within our discussion.

## 4. Discussion

The discussion section presents four themes from the findings of the literature review. These are (1) predominance of mental health studies, (2) opportunities for standardization of sensing approaches, (3) opportunities for using machine-learning advancements in eHealth research, and (4) sensing trends over the years and future scope

### 4.1. Predominance of Mental Health Studies

Over the past 5 years, the literature in this space has shown an overwhelming prominence for mental health as an area of study, with a mix of conditions such as depression [8,79,86,89], bipolar disorder [94,95], and schizophrenia [91,93]. The majority of studies explored how behavioral patterns influenced the mental health of individuals by primarily examining a range of wellbeing factors. The sheer number of works in this space indicate the scope and potential of smartphone sensing in mental health research.

On the other hand, studies focused on physical health and wellbeing, with factors such as physical activity [22,50,53] and sleep [19,57,58] being studied. Although these are not health conditions in themselves, they correlate to other conditions (e.g., reduced sociability influencing depressive symptoms). Of the one physical chronic condition covered (Parkinson’s), the emphasis was on evaluating its impact on motor symptoms such as gait and posture [28,29]. As such, the focus was primarily on physical-activity recognition. Previous works have studied the use of the camera sensor to monitor factors such as heart rate and skin diseases [115]; however, very few studies in this sample collected such data.

The fact that the numbers for general health and chronic conditions were lower than those of mental health conditions could be due to several reasons. Firstly, it is arguable that mental health conditions are a particularly important concern to measure, not only due to their prevalence over the globe [116,117], but also due to limitations that exist around actively collecting data with participants experiencing mental health concerns. For example, the subjectivity of self-reports [2,20,100], reliance on user input and compliance [61,99], and recall bias [70,81] might make passive sensing a more favorable approach to monitor mental health. Secondly, one of the limitations around smartphone sensing that was discussed was the impact of smartphone placement on physical data [2,3,11,103]. Studies monitoring factors such as sleep [19,84] and physical activity [2,11] discussed how different smartphone placements would render data from one or more sensors less reliable, thus impacting the accuracy of results. There is plausibly a higher need for accuracy when measuring physical data longitudinally [11,103], and physical symptoms or their proxies might be challenging to monitor passively through the smartphone. As such, objective monitoring of physical health conditions could potentially be better suited to wearables or smart home devices. There is significant literature looking at wearable and ambient sensing for monitoring physical conditions (e.g., [118,119,120,121,122]), which was beyond the scope of this literature review but should be considered by researchers wishing to explore chronic health conditions in the future.

### 4.2. Opportunities for Standardization of Sensing Approaches

Perhaps one of the more striking findings was the variety of different approaches employed across the literature in terms of sensing (e.g., active/passive) and the applications that were deployed for data collection. Although a small number of studies used existing frameworks and datasets [9,27,75,76,81], the majority of them developed custom applications from the ground up for their research. As such, significant time and resources would have been expended on the bespoke development of data-collection applications and analysis pipelines, unique to each individual study. Studies collecting similar types of data could benefit from using standardized approaches and existing frameworks, with the effect of conserving resources and accelerating up research.

Researchers across the globe have made significant efforts in recent years to provide open-source frameworks for development and analysis [32,33,34,113], which have already garnered numerous citations in the literature to date. Frameworks such as AWARE [123] and RADAR-base [124] have communities of practice and support available (e.g., Slack channels) and encourage people to contribute to ongoing development and feature development through open GitHub repositories. As such, there is scope for increased collaboration and innovation towards novel approaches, rather than the redevelopment of similar tools from scratch. That said, given their limited use to date, it might be the case that there are barriers to overcome around sharing resources such as these (e.g., lack of broad awareness, skill sets of research teams, or that these frameworks do not precisely fit the design of a specific study). Further exploration focusing on potential barriers towards the use of existing frameworks would be useful to understand how we might overcome these as a research community.

Besides reducing the amount of work involved, standardization could also enable deployment of the systems to wider populations in the future. The literature showed that the majority of studies used Android devices for data collection. This could partly be due to restrictions on collecting certain types of data with iOS [8,9,54,55,88], but also due to the additional overheads required to develop systems for both platforms. Using existing frameworks could potentially ease the development of multiplatform applications, enabling greater inclusion of iOS users in future work. This would ultimately reduce selection bias (i.e., by limiting the participant groups based on the type of device they use) [55] and enable increased access to participant groups.

In short, there is significant promise in this space, and increasing awareness and collaboration amongst practitioners could pave the way for standardization, and thus is an important consideration for future work.

### 4.3. Opportunities for Using Machine-Learning Advancements in E-Health Research

The effective use of machine learning (ML) is essential for studies exploring human-activity recognition (HAR) with systems using passive sensing for predicting user behavior. Many papers in the sample had some form of HAR pipeline, but there were very few that focused more on recent advances made in HAR over the last few years, such as deep-learning approaches [125] or even more advanced feature representations [126]. Whilst this might simply be due to the skillsets of the research teams, or the focus of the research questions/aims, it does show a gap in the literature that could explore the scope for implementing more modern approaches in machine learning for smartphone sensing, which could potentially increase the performance of HAR. Future work is required to investigate how we can best bring together the knowledge of multidisciplinary groups, to make use of combined expertise.

### 4.4. Sensing Trends over the Years and Future Scope

A substantial amount of research has been carried out in the space of smartphone sensing for healthcare, which has led to several systematic reviews providing an overview of the advancements at different points in time. Most previous reviews have focused on specific aspects of smartphone sensing. For example, prior works reviewed the literature with a focus on specific conditions or set of conditions (e.g., Parkinson’s [31], drug use [38], mental health [127,128], and physical activity [129]), whilst others narrowed their purview by some studying specific forms of data collection (e.g., EMAs [130], multimedia sensors [116]), and analysis approaches [40]. In contrast, our review presents a broad overview of all such factors and does not focus on specific conditions and sensing approaches.

There was, however, another complementary systematic review conducted in 2019 that presented the broader perspective of smartphone sensing [131]. They reviewed passive-sensing literature from 2014–2019 and found that the majority of the studies were examining physical activity and mental health conditions. This highlights a continuing trend of the prevalence of mental health studies. This is further showcased by the number of systematic reviews examining mental health conditions over the past decade [37,127,128,132,133,134]. While their review focuses solely on passive-sensing aspects, we have also discussed the several forms of active data collection used in the literature, which were widely used in the studies. Additionally, our review also highlights the applications and resources used in the literature, which can serve as a guide for future research.

All this said, it is crucial to consider how smartphone sensing may be impacted in the future due to changes in privacy laws and operating system restrictions on sensor data collection. Notably, there has been a significant push to protect user privacy and provide users with greater control over their data. For example, Google has made several changes to Android user permissions over the years that provide greater control over the data collected by smartphone applications. These privacy changes have impacted several factors such as application access to certain sensors, the granularity of data collected and applications collecting data in the background [46]. Additionally, changes to privacy laws will also impact data collection, storage, and sharing practices. The impact of such changes is not predictable; for example, the GDPR provides expansive opportunities to conduct research, provided certain data-protection obligations are met (e.g., data security, see [135] for a summary of relevant general obligations), yet the approach adopted by platform maintainers (e.g., Google in respect of Android, Apple for iOS) is a lot more conservative. This implies that the requirements of the privacy laws such as GDPR cannot be used to determine the level of restrictions that would be applied in the future. Such changes will have a significant impact on the potential of smartphone sensing and researchers may have greater restrictions on collecting user data in future studies.

## 5. Limitations and Future Work

In common with all literature reviews, there are criteria used for paper selection. This implies that relevant works in the space may have been excluded. Specifically, articles not focusing on smartphones may have been overlooked, even if that work in part uses them within a wider sensing infrastructure (e.g., smartphones used along with wearables and external sensors). The focus was solely on smartphones due to the additional concerns surrounding wearable sensors/sensing infrastructure. Factors such as cost, intrusiveness, ease of use, adoption by various population groups [136,137,138], etc., have an impact on the uptake of wearables. Such factors must be explored in depth when studying wearables. Considering these factors and using wider terminology would have surfaced a larger number of papers, albeit at the risk of providing too many results to be practically reviewed. This all said, a wider review might have also complicated our analysis and occluded the clear issues that we identified in this account.

Our findings also highlight several important directions for future work. First, there is a pressing need to investigate the barriers that may be impacting standardization of sensing approaches across research teams. Identifying these could pave the way for collaboration and lead to greater focus on innovation. Second, there is scope for leveraging machine-learning advancements such as deep learning in smartphone-sensing applications. Future work can explore the feasibility of using such approaches and evaluate if these can enhance the capabilities of smartphone sensing. Lastly, future studies could further explore the integration of smartphone sensing and other sensing modalities (e.g., [139]). There is also scope for such systems to contribute to user wellbeing, rather than studying it; for example, one could see such systems integrated into persuasive technology [140]. As such, there are a wide range of opportunities for future work that arise from our findings.

## 6. Conclusions

In this paper, we have presented a narrative review of smartphone sensing for health, covering papers published over the last five years. This narrative review has covered a diverse body of work that has used a great variety of different approaches and tools to monitor a broad range of health conditions. In addition to providing a map of the current state of the art, this paper has also presented a clear agenda forward for capitalizing upon this work, which highlights the need to move towards standardization and investigate potential barriers in the process. It highlights the scope for collaboration of clinical and human-activity research (HAR) communities together, which could enhance the potential of smartphone-based activity-recognition systems. We provide an overview of the advancements in smartphone sensing which could assist future researchers to quickly decide between different approaches and assist in making high-level decisions for using smartphone sensing.

## Figures and Tables

**Table 1:** Advantages and disadvantages of active sensing.

Advantages	Disadvantages
Highly customizable and can collect as much or as little data required [83]	Requires regular user input, places burden on the user. This may impact user acceptance, compliance, and retention [61,99].
Ability to collect data about conditions that cannot be sensed directly, such as mental health [16,70,95]	Self-reported data can be subjective and susceptible to bias [2,20,100]
Ability to provide additional context or complementary data to passively sensed data [11,53,57,88]	Reliance on user memory and recall, which may not always be accurate [70,81].

**Table 2:** Smartphone-sensing capabilities and application scenarios.

Sensor (S)/ Software Feature (SF)	What Does It Collect?	What Has It Been Used for?	Key Advantages (+)/Disadvantages (−)
Accelerometer (S)	Acceleration forces along x, y, and z axes of the device	It has been used to detect physical activity (such as standing, walking, running, etc.) and sedentary behavior [11,23,53]. Physical activity has also been used to infer mental wellbeing of individuals [15,67,92] (e.g., decline in physical activity impacting mental health)	+ Relatively privacy-sensitive. + Low power − Accuracy impacted by sampling rate. − Performance negatively impacted by device placement.
Ambient Light (S)	Amount of light the device is exposed to	It has been used alongside other sensors to understand the user surroundings. Studies used the data to infer when the user was asleep [13,57,58] and infer the amount of spent in the dark, which could provide an indication of mood/mental health [15,26,80]	− Only able to make very limited inferences by itself, used in conjunction with other sensors − Potentially impacted by device placement
Application usage (SF)	Information about the applications used on the device	It has been used to infer the communication behavior of users. Information such as application use time and genres of applications (e.g., social media) used provided an insight into the user’s sociability and wellbeing [55,70,92].	+ Can be used to infer a wide range of user interactions − Privacy concerns depending on what information is captured.
Battery status (SF)	Indicates the phone charging status (on/off)	It was used as a proxy measure to infer phone-usage behavior. For example, studies monitoring sleep used it as an indicator of the person sleeping, assuming they charge their phone overnight [19,57].	+ Privacy-sensitive − Only able to make limited inferences by itself, used in conjunction with other sensors
Bluetooth (S)	Information about nearby Bluetooth-enabled devices	It has been used to infer the sociability of the user. By collecting information such as count of nearby Bluetooth devices, number of recurring devices etc., studies were able to infer the social context of users [9,61,76].	− Not all nearby devices may have Bluetooth turned on
Camera (S)	Capture images and videos	It has been used to infer the user’s emotions by capturing facial images [71]. Another study used the camera to capture eye-movement data and checked if such features could provide an indication of the user emotions [74].	+ Ability to visually monitor user behavior − Higher impact on battery life − Relatively serious privacy concerns, due to video recording.
Global Positioning System (GPS) (S)	Latitudinal and longitudinal coordinates indicating physical location	It has been used to infer the mobility of a user (number of places visited, time spent outdoors, time spent at home) which has an impact on wellbeing [26,27,84] (e.g., too much time spent at home indicating a decline in sociability and in turn mental health [7])	+ Can use location to make a wide range of inferences about behavior and wellbeing. − Higher impact on battery life compared to other modes of sensing. − Privacy concerns, especially when used with a high degree of granularity.
Gyroscope (S)	Rotational forces along the x, y, and z axes of the device	It has been used in conjunction with the accelerometer for activity recognition. Assisted in detecting activities such as walking, standing, laying etc. [11,30,49]	+ Can increase recognition accuracy compared to an accelerometer alone, due to the provision of additional rotational information. + Low power − Impacted by device placement
Microphone (S)	Collect audio recordings from the surroundings	It has been used to infer surrounding sound, which can provide information about the user’s context. Some studies used it to detect if the user was alone (i.e., sociability) by listening for conversation [3,54,84]. Some used it to detect if the user was sleeping if the surroundings were quiet (along with other sensor data such as light) [57,58].	+ Has utility in respect of social sensing. − Impacted by device placement − Relatively serious privacy concerns due to audio recording.
Phone lock/unlock status (SF)	Indicates whether the phone is locked or unlocked	It was used to infer phone usage behavior. By calculating the time between the unlock and lock states, studies estimated the phone usage time [24,25,91]. Additionally, this was also used as one of the factors to infer sleep (i.e., phone in locked state for long time during bedtime hours) [57,58,91]	+ Privacy-sensitive. − Unreliable by itself, used in conjunction with other sensors
Phone-call and text-message logs (SF)	Logs/records of text messages and phone calls	It has been used to infer the communication patterns of users, which correlate to social wellbeing. For example, decreased frequency of such communication features could indicate decreased sociability of individuals [55,69,85]	− Privacy concerns depending on what information is captured.
Screen status (S)	Indicates screen on/off status	Similar to phone lock/unlock status, it was used to infer phone-user behavior. Screen on/off indicated when the device was being used, which could further indicate distracted/anxious behavior [84], or infer sleep [19,91]	− Unreliable by itself, used in conjunction with other sensors − Can be impacted by phone notifications (resulting in screen on state)
Wi-Fi (S)	Indicates nearby Wi-Fi connectivity	These types of data were used as a complimentary source to infer location and indicated indoor mobility [8,51,60,88]	+ Can increase accuracy of location determination

**Table 3:** Smartphone-sensing capabilities and application scenarios (Data as of April 2022).

Name [Original Ref]	Platforms Supported	Codebase	Last Updated (Year)	Cited by
AWARE [32]	Android, iOS	Android: https://github.com/denzilferreira/aware-client (accessed on 1 May 2022) iOS: https://github.com/tetujin/aware-client-ios-v2 (accessed on 1 May 2022)	Android: 2020 iOS: 2021	[12,62,75,79]
Beiwe (Both open-source and Software-as-a-Service (SaaS) framework for data collection and analysis) [33]	Android, iOS	https://github.com/onnela-lab (accessed on 1 May 2022)	Android: 2021 iOS: 2022	[9,50]
EARS (Initially open-source, now available as SaaS for data collection and analysis [34,111]) [34]	Android, iOS	https://github.com/C4DMH (accessed on 1 May 2022)	Android: 2020 iOS: 2020	[78]
Emotion Sense [112]	Android	https://github.com/emotionsense (accessed on 1 May 2022)	2017 Project is no longer maintained	[8,88]
RADAR—base [113]	Android, iOS	https://github.com/RADAR-base (accessed on 1 May 2022)	Android: 2022 iOS: 2021	[7]

## Data Availability

Not applicable.

## Abbreviations

Acronym	Meaning
GPS	Global Positioning System
HAR	Human Activity Recognition
NLR	Narrative Literature Review
PRISMA	Preferred Reporting Items for Systematic Reviews and Meta-Analyses
PHQ	Patient Health Questionnaire
UCLA	University of California, Los Angeles
EMA	Ecological Momentary Assessments
Wi-Fi	Wireless Fidelity
ML	Machine Learning
